# HPV-based screening for cervical cancer among women 55-59 years of age

**DOI:** 10.1371/journal.pone.0217108

**Published:** 2019-06-14

**Authors:** Lovisa Bergengren, Gabriella Lillsunde-Larsson, Gisela Helenius, Mats G. Karlsson

**Affiliations:** 1 Dept of Women’s Health, Faculty of Medicine and Health, Örebro University, Örebro, Sweden; 2 Dept. of Laboratory Medicine, Faculty of Medicine and Health, School of Health Sciences, Örebro University, Örebro, Sweden; 3 Dept of Laboratory Medicine, Faculty of Medicine and Health, Örebro University, Örebro, Sweden; Istituto Nazionale Tumori IRCCS Fondazione Pascale, ITALY

## Abstract

**Aim:**

Many cervical cancers occurs among women over 65 and prevalence of HPV genotypes in this age cohort is sparingly studied. One aim of this study was to study the prevalence and distribution of HPV genotypes in women 55–59 years, with normal cytology when exiting the screening program. Secondly, HPV clearance as well as the value of HPV genotyping and/or liquid based cytology as triage tests for identifying histological dysplasia among women with persistent HPV was studied.

**Methods:**

Women that exited the screening program with normal cytology, between the years 2012–2014, in Örebro County, Sweden, were invited to this study. A total of 2946 samples were analyzed with a broad-spectrum assay to detect both hrHPV and lrHPV in order to investigate the distribution of genotypes. In the consent group, women with a positive hrHPV test were offered a follow-up test and a cone biopsy for histological confirmation, and a follow up sample 6 months post cone.

**Results:**

The overall prevalence of hrHPV was 7.4% and 59% of them remained hrHPV positive in a follow-up test after 12 months. A total of 99 women had a cone biopsy done, where 19% showed histological dysplasia. HPV 53 was the most common genotype, and among women with histology confirmed LSIL or HSIL, HPV 31 was most common. A positive hrHPV result showed a PPV of 25% for LSIL+ and 12.5%for HSIL+. Using detection of HPV 16/18 genotypes as a triage test for hrHPV positive tests, indicated FNR for histological LSIL+ and HSIL+ of 94% and 87.5% respectively, whilst triage based on cervical cytology had a FNR of 69% for LSIL+ and 37.5% for HSIL+.

**Conclusion:**

The most common hrHPV genotypes among women 55–59 years of age were non HPV16/18 genotypes, and in this population, these genotypes represented most of the histological verified HSIL lesions. This result does not support the proposition of a HPV 16/18 triaging test after a positive hrHPV test as a marker of histological HSIL+ cervical lesions in women over 55 years of age. Similarly, cytological triage after a positive hrHPV showed no additional benefit in this population. Specific triaging tests should be validated to follow post-menopausal women with a positive hrHPV test.

## Introduction

A national screening program for cervical cancer was introduced in Sweden around 1965 and since then the incidence of cervical cancer has been markedly reduced till around one third [1, 2]. Obviously, the incidence has only declined among the group of women included in the screening program, which until recently, was women between 23–60 years of age, where smears were recommended every third year until 49 years and every fifth year from the age of 50. Data show however that a large part of the newly diagnosed cervical cancers are found among women over 65 years of age and/or women who have not participated in the screening program for the last 7 years [3, 4]. These cancer cases are also detected at a more advanced stage and thus having a worse prognosis with more fatal outcomes [3].

Until recently, cytology based screening was the standard both nationally and internationally but in Sweden and other countries, screening programs are transferring from primary cytology to detection of human papilloma virus (HPV) at least for parts of the population [5]. The Swedish guidelines adopted in 2015, are based on data showing that primary HPV test has higher sensitivity for detecting high-grade squamous intraepithelial lesions (HSIL) compared to cytology alone [6, 7]. Widely accepted are 14 high-risk HPV (hrHPV) used for HPV screening internationally (HPV 16, 18, 31, 35, 39, 45, 51, 52, 56, 58, 59, 66, 68),[8], but there are other HPV types that are classified as probably and possibly hrHPV [9]. HPV 16 and 18 together cause 70% of all cervical cancers [10–13].

The prevalence of HPV in women exiting the screening program is sparingly studied. Data indicate that there are variations of HPV genotypes and prevalence of HPV in different age cohorts among precancerous lesions [13–15]. Other observations show that the prevalence of hrHPV among older women is higher than among women 35–45 [14, 16, 17] or the expected fall by age seen in many studies is plateauing in age 50–60 and thereafter falling [18]. Reasons for this are discussed, stating higher number of new infections [19] or reactivation of non-detectable persistent infections [20, 21]. At the same time studies indicate that women are sexually active a longer period of lifetime [22].

Other data suggest that postmenopausal women are less likely to acquire new infections since the transformation zone and the squamocolumnar junction is retracted when reaching menopause [23, 24]. Also at menopause, the epithelia in the cervix and vagina becomes atrophic [25]. How these changes influence the results in the HPV tests in the screening program is not well known.

Population based prevalence data with a broad-spectrum assay of high-risk HPV and possibly and probably hrHPV among peri-/postmenopausal women were missing until recently. Different studies show a prevalence that differ greatly worldwide, and among European studies HPV prevalence among women between 55–60 differ from just below 5% to just over 10% [13, 18, 21, 26–28].

Therefore, the aim of this research was to study the prevalence of HPV and the distribution of genotypes in an age specific cohort, women 55–59 years, with normal cytology when exiting the screening program. hrHPV positive women were followed-up in order to investigate if certain HPV genotypes and/or cytology could predict cervical histological changes in this particular age group, in order to modulate screening and follow-up strategies among postmenopausal women.

## Material and method

### Study design

**Fig 1:**
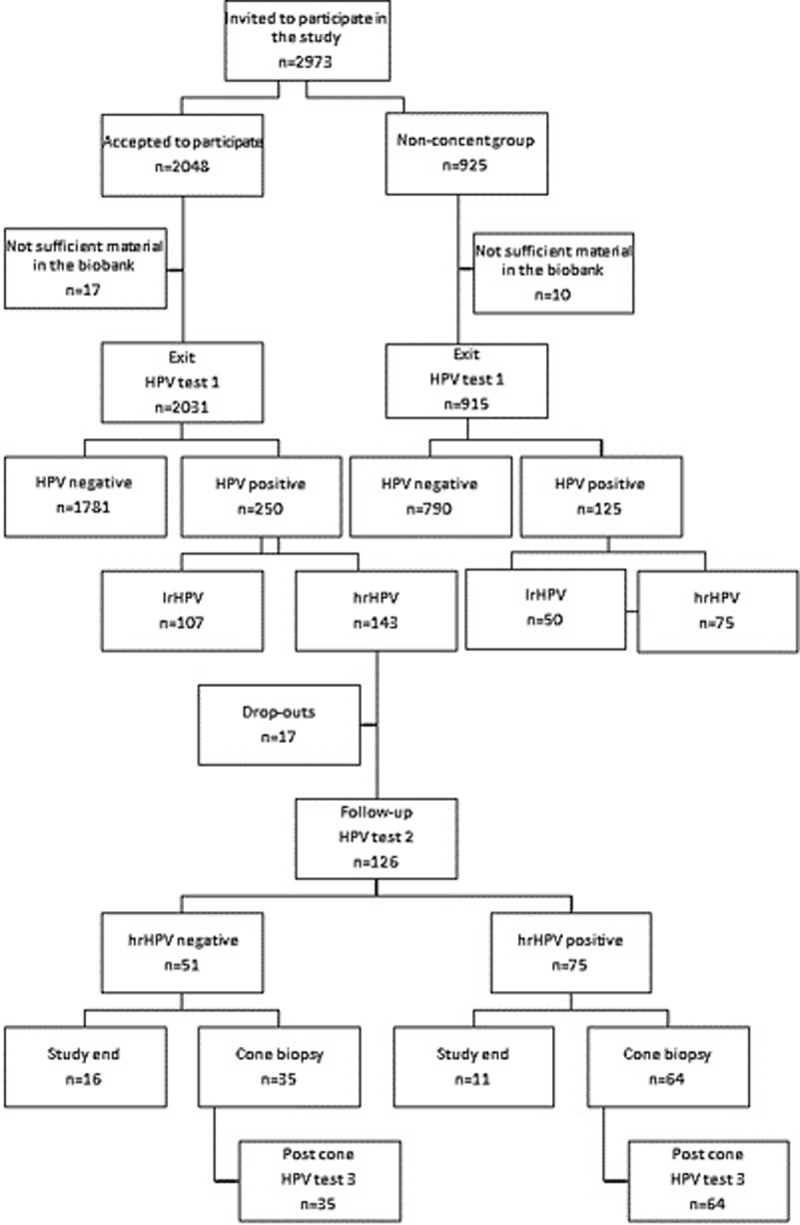
Flowchart over study design. hrHPV are defined according to IARC group 1, 2A and 2B.

A total of 2973 women in Örebro County, Sweden, that had their exit samples from the screening program during 2012–2014 in the biobank, were invited to participate in this study. Of those, 2031 women signed the consent and had sufficient cell material to analyze. Additionally, 915 women did not respond to the invitation (non-consent group) but had samples in the biobank [29] available for HPV test (Fig 1). During the years 2012–2014 the attendance rate in the screening program in Örebro County among this age group was 71%. The liquid based cytology (LBC) samples (ThinPrep, Hologic, Marlborough, MA, USA) were withdrawn from the biobank and analyzed for HPV with a DNA-based assay detecting 35 HPV genotypes, both low risk HPV (lrHPV) and hrHPV.

For DNA extraction, 200 μl biobanked sample (concentrated LBC) or 1000 μl LBC sample was used for extraction with QiaAmp DNA mini kit (Qiagen, Hilden, Germany) [30]. CLART^®^ HPV2 (Genomica, Madrid, Spain) was used for genotype determination. This test detects HPV genotypes in International Agency for Research of Cancer (IARC) classification high risk group 1^§^2A^§§^ (probably high risk) and 2B^§§§^, (possibly high risk) and several lrHPV. The assay targets the L1-region of the virus and 35 different genotypes (HPV6, 11, 16^§^, 18^§^, 26^§§§^, 31^§^, 33^§^, 35^§^, 39^§^, 40, 42, 43, 44, 45^§^, 51^§^, 52^§^, 53^§§§^, 54, 56^§^, 58^§^, 59^§^, 61, 62, 66^§§§^, 68^§§^, 70^§§§^, 71, 72, 73^§§§^, 81, 82^§§§^, 83, 84, 85^§§§^ and 89) as well as the human gene *CTFR*.

PCR-reactions were run in 50 μl reactions containing 45 μl CLART HPV2 Amplification kit (Genomica) together with 5 μl of DNA. The low-density microarray platform, CLART^®^ (Clinical Array Technology) was used for detection of PCR-product and results were analyzed automatically in software CLART^®^ Human Papillomavirus 2 specific software (Genomica). Prevalence of HPV was analyzed in both the consent group and anonymously in the non-consent group. Here after, in the following text when referring to “hrHPV”, genotypes according to IARC class 1, 2A and B are meant [9]. “hrHPV with clinical significance” are 14 genotypes (HPV 16, 18, 31, 35, 39, 45, 51, 52, 56, 58, 59, 66, 68) widely used and accepted for cervical cancer screening [8]. “lrHPV+hrHPV” are the 35 genotypes included in the assay used for genotyping in this study, the hrHPV IARC class 1, 2A and B and lrHPV 6, 11, 40, 42, 43, 44, 54, 61, 62, 71, 72, 81, 83, 84 and 89.

In the consent group, women positive for hrHPV in their exit sample were invited for a follow-up visit with new professional sampling. Time between the exit samples and follow-up samples varied between 7 to 39 months, mean 24 months. Additionally to the follow-up sampling, they were offered an appointment to a gynecologist for loop electrical excision procedure for a cone biopsy. The last follow-up sample in the study was 6 months after the cone biopsy (Fig 1). None of the women in the study were vaccinated with HPV vaccine.

Excel 2010 (Microsoft, Redmond, WA, USA) and SPSS version 22 (IBM, Armonk, NY, USA) were used for data collection and evaluation. Chi Square test was used for calculations of significance, P<0.05 was considered statistically significant. Positive predictive value (PPV) and negative predictive value (NPV) were also calculated, as well as false negative rate (FNR), for calculations comparing test results between methods improperly indicating no presence of dysplasia, when the histological exam identifies dysplasia.

### Morphological assessment

ThinPrep cytology slides were assessed by experienced and IAC certified cytotechnicians and classified according to present guidelines [31]. Concerning histopathology, cone specimens were formalin fixed, paraffin embedded and thereafter slides were cut at 4 μm and stained with hematoxylin and eosin and evaluated according to present WHO classification [32], by either of two senior pathologists. Furthermore, the presence or absence of the transformation zone as well as the presence of dysplasia in the surgical margins were noted [24].

### Ethical approval

This study was approved by the regional ethics committee board in Uppsala (Dnr 2014/122). All women included in the study have signed an informed consent before the exit sample was analysed and the invitations for further follow-up were sent out. The ethical approval also contained an agreement to analyse exit samples anonymized in the non-consent group.

## Results

### HPV prevalence and distribution of genotypes in the exit samples

Among the 2973 women who exited the screening program, 2946 HPV tests were performed including 2031 women who accepted to participate in the follow-up study and 915 women who did not and whose HPV tests were analyzed anonymously. Median age (56,3 years) and HPV genotypes did not differ statistically between the two groups (Table 1). The prevalence of lr- and hrHPV together was 12.7% and prevalence of total hrHPV genotypes according to IARC group 1, 2A and B, was 7.5% (Table 1). If only considering the 14 hrHPV genotypes used in most clinical assays, the prevalence was 5.5%.

**Table 1:** Prevalence of HPV.

Groups of genotypes analyzed	All invited	Consent	Non-consent	p-value
1.lr+hrHPV, 35 genotypes	12.7%	12.4%	13.7%	0.31
2.hrHPV, 20 genotypes	7.4%	7.0%	8.2%	0.27
3.hrHPV of clinical significance, 14 genotypes	5.5%	5.2%	6.1%	0.29

*lr = low risk, hr = high risk

**Fig 2:**
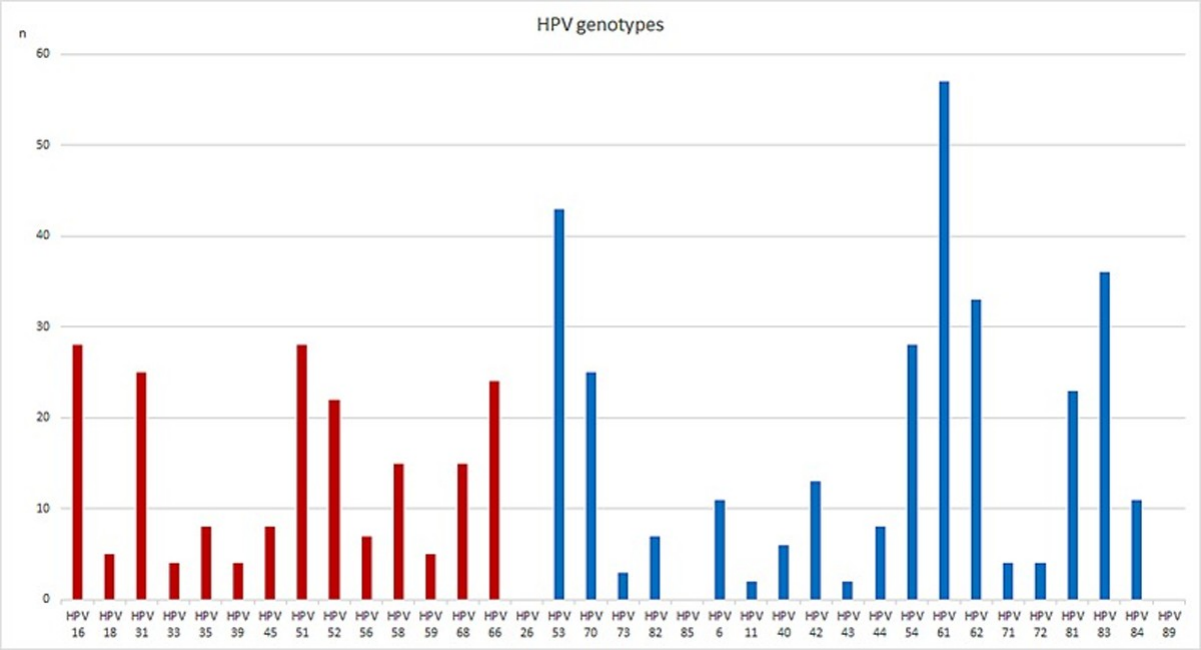
Distribution of all detected genotypes, lrHPV and hrHPV, in both the consent and non-consent group. Shown in the blue square are the genotypes included among the 14 most clinically used genotypes.

The most common hrHPV genotypes among the positive results were HPV53 (11.5%), followed by HPV51 (7.5%) and HPV16 (7.5%). Among the lrHPV genotypes HPV61 (15.2%) was most prevalent (Fig 2).

Multi infections with lrHPV and hrHPV were seen in 25.9% of the positive samples. When analyzing only hrHPV according to IARC group 1, 2A and B, 11.7% were multi infections, and when hrHPV of clinical significance were analyzed the occurrence of more than one HPV infection was seen in 8.3%.

### HPV follow-up of hrHPV positive women

Among the 143 women with positive hrHPV (group 1, 2A and B) test at exit, 126 (88%) continued with a follow-up test with a median interval between the first HPV test and the follow-up test of 24 months (range 7–39 months). If applying 12 months as the time range for clearance, 113 individuals had a follow-up of 12 months or more, 67 out of them had still a HPV infection at follow-up, thus the clearance rate at 12 months or more was 59%. Including all 126 even if tested before 12 months the clearance rate was the same, 75/126 (59.5%). If only focusing on the 14 hrHPV genotypes of clinical significance 48/84 (57%) were still hrHPV positive after 12 months or more.

Concerning single and multi-infections of the 126 women continuing the study, 101 had HPV infection with a single HPV genotype. In this group, HPV clearance was observed for 43 women (42.6%) with a median time interval between exit test and follow-up of 26 months. At the exit test (HPV test 1), 25 out of 126 women were testing positive for multiple hrHPV genotypes. Among these, 8/25 (32%) had a negative HPV test at follow-up, with a median time interval of 19 months, thus there was no difference in clearance rate between single and multiple infections (Chi^2^ 0, 93, p = 0,335).

**Fig 3:**
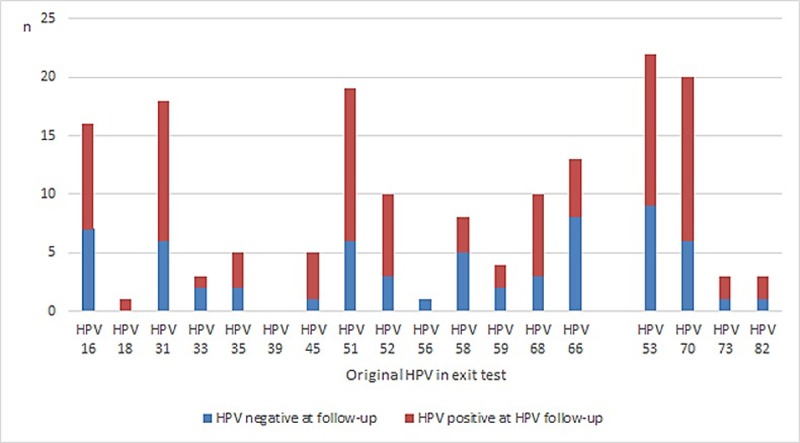
Comparison of original HPV present in the exit test, amongst follow-up tests negatives or positives.

Concerning the 75 hrHPV positive cases at follow-up, 15/75 (20%) were multi infections. The majority, 71/75 had the same or partly the same genotype, for example, one of the genotypes in a multi infection was not detected in the second test (Fig 3).

### Cytological follow-up

Out of the 64 hrHPV positive women continuing in the study to the cone biopsy procedure, 13 cytology samples showed pathological cytology; 6 atypical squamous cells of undetermined significance (ASCUS), 3 low-grade squamous intraepithelial lesions (LSIL), 3 HSIL and one sample atypical glandular cells (AGC), when analyzing cytology on repeated test. Both cytology positive samples and histology abnormality was seen in five samples (Table 2). Out of the 35 hrHPV negative women continuing in the study to the cone biopsy procedure, the repeated test showed pathological cytology in 3 samples, all ASCUS.

**Table 2:** Results of hrHPV analysis at follow-up testing (7–39 months after exit sample) with histopathological correlation. Triage data concerning both genotyping as well as cytology is also included in the hrHPV positive group. (AGC = atypical glandular cells, ASCUS = atypical squamous cells of undetermined significance).

	N	Triage test for hrHPV+	Triage test, n	Normal histo	LSIL histo	NPV ≥LSIL (%)	PPV ≥LSIL (%)	FNR ≥LSIL (%)	HSIL histo	NPV ≥HSIL(%)	PPV ≥HSIL(%)	FNR ≥HSIL(%)
**hrHPV neg**	35			32	3	91	25	16	0	100	12.5	0
**hrHPV pos**	64			48	8				8			
		HPV 16/18+	9	8	0	73	11	94	1	87	11	87.5
		Aberrant cytology	13	8	0	78	38.5	69	5	94	38.5	37.5
		ASCUS	6	5	0				1			
		LSIL	3	2	0				1			
		HSIL	3	1	0				2			
		AGC	1	0	0				1			

*NPV = negative predictive value, PPV = positive predictive value, FNR = false negative rate

### Histological end point

**Fig 4:**
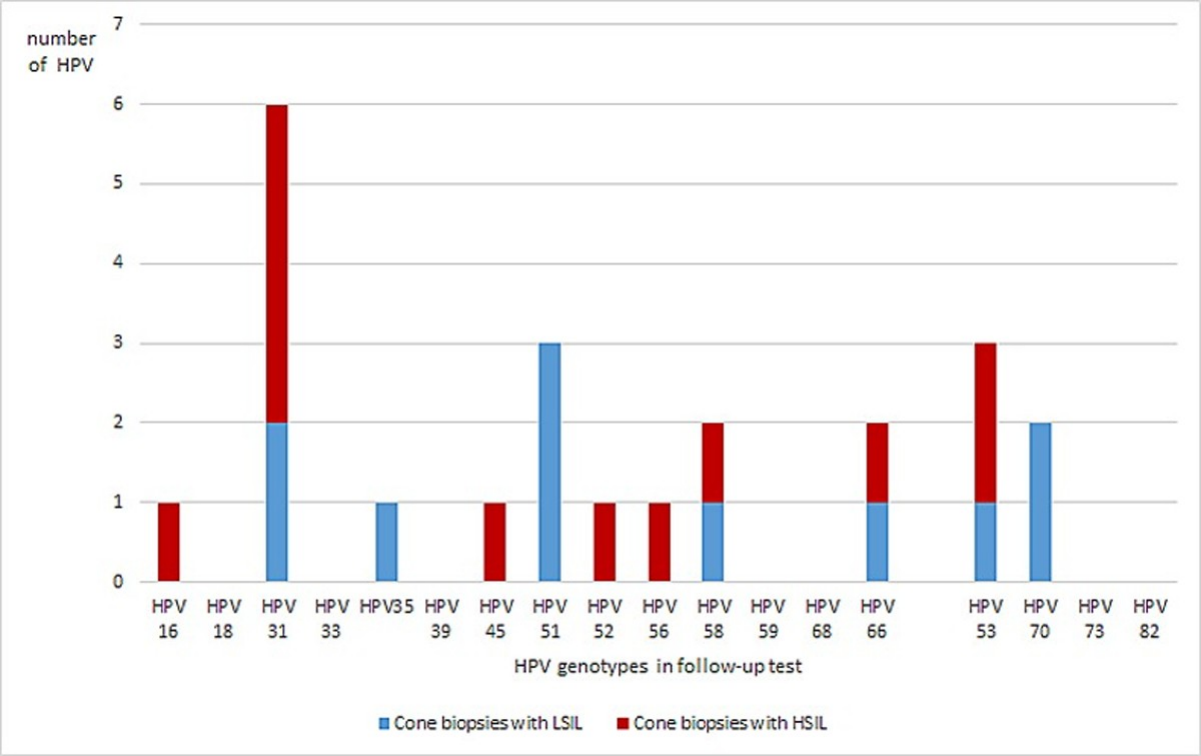
Distribution of hrHPV genotypes in the follow-up test, among the patients with ≥LSIL. Follow-up test showed multi-infection prior to six of the cones, and nine women were cleared from hrHPV at follow-up before cone biopsy.

Of the 126 women that were hrHPV positive in their exit sample, 99 went through with a cone biopsy. Of the women hrHPV positive at follow-up test, 64/75 had a cone biopsy done, and 35/51 among the hrHPV negative women (Fig 1). Histological dysplasia was detected in 19/99 (19%) cone biopsies, 11 had LSIL and 8 HSIL (Table 2). All HSIL were detected among the hrHPV positive group (Table 2). When analyzing genotypes, in the follow-up sample, among the patients with histologic LSIL and HSIL, HPV31, 51 and 53 were the most common genotypes. When only analyzing histologic HSIL, HPV31 and 53 were most common (Fig 4). Analyzing the genotyping results as to non 16/18+ and 16/18+ more HSIL were found relating to non16/18+ infections (Table 2).

### Post cone HPV test

Out of all 99 women with cone biopsies done, 33 (33%) post cone tests were still positive for hrHPV, 6 months after the procedure, 21/33 (64%) of these with the same genotypes in all samples. Interestingly, five individuals were positive with the same hrHPV genotype in the exit sample (HPV test 1) and post cone sample (HPV test 3), but the follow-up samples (HPV test 2) were negative. None of these five cone biopsies with hrHPV of the same genotype in test 1 and 3 had histological changes and the genotypes in those samples were HPV 16, 53 (n = 2), 66 and 70.

Of the 19 cone biopsies with ≥LSIL, 10 were still hrHPV positive in the post cone sample (HPV test 3) and eight of these with the same genotype. None of these had cytological abnormalities.

## Discussion

HPV-based screening has the potential to prevent additional cases of cervical cancer in women over 60 years of age. However, the clinical management of HPV positivity in this age group is not well established due to a number of reasons.

This is an age group where HPV prevalence and distribution of genotypes are mainly unknown. In this study, HPV status was assessed in biobanked samples from women exiting the screening program with normal liquid based cytology at 55–59 years, in a population based cohort. The HPV prevalence was 12.7% when including 35 both lr- and hrHPV genotypes, 7.7% when including hrHPV according to IARC class 1, 2A and 2B and 5.5% when including the 14 genotypes that are most clinically used for screening purposes. The latter in concordance with two other recent Swedish studies that showed incidence rates of 5.6 and 5.5% respectively [33, 34], as well as and in the Athena trial [18] with women in the same age group. The most common genotype in our study was HPV 53 in IARC group 2B, 11.5%, whilst HPV 16 constituted only 7.5% of the hrHPV genotype findings [14, 17, 26]. Even though these data are from a group with normal cytological finding it is interesting to note the large contribution of hrHPV of non HPV16/18 genotypes both in initial “exit sampling” and at follow-up as well as in the histologically confirmed HSIL.

Persistence of HPV infection is often referred to as a positive test after 12 months internationally, even though there is still no consensus concerning the definition of persistency of HPV infections. In this study 67/113 (59%) remained hrHPV positive after 12 months. Looking at our data of type-specific clearance rates between exit test and follow-up test, with the obvious limitation of low case numbers, the clearance rate for HPV16/18 and non-HPV16/18 were similar, 41% (7/17) vs 40% (44/109), with no statistical difference (Chi^2^ 0.004, p = 0.95). Age is shown to be a risk factor for persistence and among younger women, most infections clear [35].

Our data show no statistical difference in HPV clearance between HPV multi infections (32% clearance between exit and follow-up sample) as compared to single infections (43% clearance). To the best of our knowledge, there is no understanding of the clinical meaning of multiple infection in the genital tract and consensus is pending on whether a multiple infection leads to higher risk for cervical neoplasia [36–39]. Due to the small groups in this study, data cannot confirm neither deny multi-infections role in persistence or not.

It is well known that HPV as primary screening is the superior test in women, at least over 30, and is recommended by WHO, EU and the Swedish National Board of Health and Welfare. Initially screening was performed with cytology PAP smear, but cytological screening of women aged 60–69 ceased in Sweden during the 1990’s due to the limited sensitivity and specificity of PAP smears in this age group [40]. However LBC, the current method for cytology, has only limited data concerning performance in the same age group (postmenopausal women) [41]. From our limited data comparing histopathological findings with LBC at the follow-up sampling, the value of LBC in terms of performance to detect HSIL, using cone biopsies as the reference method, is poor and must be considered non-adequate in the clinical context. We consider the use of cone biopsies as the reference test comparing both cytology and HPV analysis as the best available method compared to studies using cervical biopsies, in this age group. Thus performing a cone biopsy on hrHPV positive elderly women might be the only way to identify LSIL and HSIL and prevent further disease progression since it is difficult to perform an accurate colposcopy with biopsies in this age cohort as well [42].

HPV genotyping of HPV16/18 as an augmented screening tool among women with hrHPV and normal cytology/LSIL have been proposed [11, 43] and debated [44]. Even though data in younger cohort of women support this approach, our data do not support such an approach among elderly women because, in our limited cohort, non-HPV16/18 HSIL constitutes 87.5% of histological verified HSIL. Using HPV 16/18+ for triage of hrHPV positive test, we found a FNR of 87.5% for HSIL+ histological lesions, while cytology had a FNR of 37.5% suggesting limitations of both tests as triaging tools for older women with positive hrHPV tests.

Usually HSIL is the endpoint in studies but maybe a LSIL in this age cohort with a persistent hrHPV infection is as accurate to identify? Another interesting finding is the hrHPV positive LSIL group among the cytological normal women. None of these cases were identified by cytology or would have been detected by HPV16/18 as a supplementary tool. Furthermore, the progression from LSIL to HSIL varies in time [45] and the natural regression/progression rate among elderly (>60 years) women with LSIL is, as far as we know, not studied. The long time risk for a LSIL lesion in a group of women with a life expectance of > 20 years motivates the demand on a screening setting to identify these women.

Post cone tests, 6 months after the procedure were still positive for hrHPV in 33%, even though an adequate cone biopsy including the transformation zone had been conducted. A number of different mechanisms can be in place, persistence, reinfection and reactivation of latent infections among them, or the possibility that the test is taken to close in time to the cone biopsy. In this study we have followed the Swedish national guidelines where a “test of cure” (post cone test) is recommended 6 months after the biopsy.

Thus, our data addressing the question of how a hrHPV woman in the postmenopausal age group should undergo follow-up indicates that there is a need for different strategies in different age groups. A non-interventional approach on the initial hrHPV finding still seems to be valid since no HSIL occurred in the HPV negative group at follow-up, but what is the best time-interval for the follow-up of women over 55 years with a normal cervical smear and a positive hrHPV test? Furthermore, neither cytology nor HPV 16/18 complementary analysis of the HPV persistent group could be used in order to limit the number of surgical procedures since major pathological findings occurred among women with normal cytology and/or nonHPV16/18 at follow-up. Maybe the most interesting finding is the prevalence and significance concerning HSIL lesions of IARC group 2A/B genotypes, especially HPV 53 in our study of elderly women. Thus, HPV test with broad genotype coverage and liberally use of cone biopsies would be key components in such a follow-up schedule.

Altogether, our data highlights the need for a solid evidence based guideline for the clinical management of hrHPV positive women intended to exit the screening program where the concomitant cytological evaluation is normal. Further studies to identify biomarkers such as for example methylation, in the context for pretreatment persistence and/or clearance as well as further information of sexual habits and partner HPV testing must be taken into consideration but are presently out of scope of our present study.
